# Comparative Antimicrobial Resistance and Prevalence of Methicillin Resistance in Coagulase-Positive *Staphylococci* from Conventional and Organic Dairy Farms in South Korea

**DOI:** 10.3390/antibiotics13070617

**Published:** 2024-07-02

**Authors:** Therese Ariane N. Neri, Hyunjung Park, Sujin Kang, Seung Hee Baek, In Sik Nam

**Affiliations:** 1 School of Animal Life Convergence Science, Hankyong National University, Anseong-si 15759, Republic of Korea; taneri@hknu.ac.kr (T.A.N.N.); joy1392@naver.com (H.P.); jiny@hknu.ac.kr (S.K.); 2 Research Center for Environmentally Friendly and Quality Livestock Production Technology, Hankyong National University, Anseong-si 15759, Republic of Korea; shbaek@hknu.ac.kr; 3 Institute of Applied Humanimal Science, Hankyong National University, Anseong-si 15759, Republic of Korea

**Keywords:** *Staphylococcus aureus*, coagulase-positive *Staphylococci*, methicillin-resistant *S. aureus*, antimicrobial resistance, bovine mastitis, organic dairy farm, conventional dairy farm

## Abstract

Bovine mastitis (BM) has caused huge economic and financial losses in the dairy industry worldwide, with *Staphylococcus aureus* as one of its major pathogens. BM treatment still relies on antibiotics and its extensive use often generates methicillin-resistant *S. aureus* (MRSA) and mupirocin-resistant *S. aureus* (MuRSA). This study compared the antimicrobial resistance trend in coagulase-positive *Stapholococci* (CoPS) isolated from BM milk in conventional and organic dairy farms and checked prevalence of MRSA and MuRSA. A total of 163 presumptive *Staphylococci* were isolated, wherein 11 out of 74 from 4 conventional farms (CF1, CF2, CF3, CF4) and 17 out of 89 from 3 organic farms (OF1, OF2, OF3) exhibited coagulase activity. Multiplex-PCR amplification confirmed at least one coagulase-positive isolate from CF1, CF2, CF3, CF4, and OF1 as *S. aureus*, denoted by the presence of the *nuc* gene. Three isolates from CF2 contained the *mecA* gene, indicating MRSA prevalence, while the MuRSA gene marker, *mupA*, was not detected in any of the isolates. Antimicrobial testing showed that conventional farm isolates were more resistant to antibiotics, especially ampicillin and tetracycline. This suggests a risk of developing multidrug resistance in dairy farms if antibiotic use is not properly and strictly monitored and regulated.

## 1. Introduction

Bovine mastitis (BM) is one of the major concerns in the dairy industry, causing considerable economic and financial losses worldwide [1]. BM is the inflammation of mammary glands caused mainly by bacteria, fungi, some microscopic algal species, viruses, and occasionally by physical trauma [2]. BM’s pathogenic agents consist of different of Gram-positive and Gram-negative bacteria, and are classified as contagious (e.g., *Staphylococcus aureus*, *Streptococcus agalactiae*, *Mycoplasma* spp., *Corynebacterium bovis*) or environmental (e.g., *Escherichia coli*, *Enterococcus* spp., coagulase-negative *Staphylococcus*, *Streptococcus uberis*) [1,2]. Contagious pathogens live in the cow’s udder and teat skin and grow and colonize at the teat canal. Mastitis caused by contagious pathogens is often characterized by subclinical intramammary infections with no visible signs but with increased somatic cell count, and can be transmitted from cow to cow, particularly during milking [3,4]. On the other hand, environmental mastitis pathogens are found in the cow’s environment and can enter the teat during milking or when the cow’s inherent or natural immunity becomes weak. Environmental pathogens are opportunistic microorganisms that cause clinical mastitis, which can be easily noticed and detected, as characterized by evident swelling of the teat and with occasional pus included in the milk [2]. And among these pathogenic agents, the most related to both clinical and sub-clinical mastitis is *S. aureus* [5], which has also been noted to cause several human infections and, in turn, raises the concern of cross-infection between humans and animals through contaminated food and other factors. Although the risk of contracting MRSA through pasteurized milk consumption is low, raw milk and milk products may still pose a threat to the public when exposed to viable bacteria [5,6].

The general measures applied to reduce infection and prevent the spread of mastitis include improving sanitation (e.g., enhanced milking hygiene), teat disinfection after milking, and maintenance of milking machines [2,3]. However, these protocols can only reduce the degree of contamination and do not necessarily eliminate the existing infection. Treatment of active mastitis still relies greatly on antibiotics [5,6]. The continuous and extensive use of different antibiotics causes some bacterial strains like *S. aureus* to become resistant against antimicrobial agents, particularly on β-lactams. Hence, the prevalence and rise of *S. aureus* strains, coagulase-positive *Staphylococci* (CoPS) spp., coagulase-negative *Staphylococci* (CoNS) spp., and other bacterial strains that have developed resistance to different antibiotics such as penicillin, clindamycin, tetracycline, methicillin, mupirocin, and vancomycin over the years [5,6,7,8,9], particularly in conventional farms. Consequently, organic farming has been introduced to combat the emergence of antibiotic-resistant mastitis pathogens. In organic livestock production, non-therapeutic antibiotics and growth promoters are prohibited. Antibiotic use, which is very seldom, is allowed only when the safety of animal is endangered [10]. For example, if a disease outbreak threatens the cattle’s safety in an organic dairy farm, the infected animal is isolated. If alternative treatments are ineffective, the farmer may then use antibiotics for treatment. Following treatment, the treated animal remains isolated for a specific period to ensure that no antibiotic residues remain in its system. Only then is the animal reintroduced to the farm’s production area [10]. However, there are still some instances where antibiotic-resistant microorganisms are found in organic farms as well [11].

In Korea, sales of antibiotics used in livestock doubled from 55.1 tons in 2011 to 96.4 tons in 2020 with the resistance level of livestock-isolated *S. aureus* for penicillin, ampicillin, and oxacillin to be 69.4%, 57.7%, and 2.3%, respectively [12] (pp. 8, 60). This growth in sales can be correlated to antibiotic use on livestock and the reported resistance level being above 50%, thus, poses a concern for investigation.

Moreover, consumers are becoming more health conscious and are favoring “organic” or naturally sourced foods. Increased awareness and concern for food safety and health issues is driving the consumers to choose high-quality products, often labeled as “organic”, “antibiotic-free”, and “animal welfare-approved” milk products. Organic milk sales in Korea increased significantly from KRW 5 billion in 2008 to KRW 104 billion in 2020 [13]. With the increasing demand for the organic livestock product market, there is a need to check and validate the safety of said products.

As far as the authors know, only a few studies had been conducted that focus on comparing the antimicrobial resistance of bacterial isolates from conventional and organic dairy farms, especially in South Korea. Most of these studies have relied on bulk tank milk samples. In this study, we focused on isolating coagulase-positive *Stapholococci* (CoPS) species, especially *S. aureus*, from pooled milk samples of cattle already diagnosed with mastitis (clinical and subclinical) from conventional and organic farms in South Korea. We checked the corresponding antimicrobial resistance and investigated the prevalence of MRSA and/or MuRSA among these isolates.

## 2. Results

### 2.1. Presumptive S. aureus Isolates from Bovine Mastitis Milk Samples in Conventional and Organic Dairy Farms

Following the Korea Food Codex [14], 163 potential *S. aureus* isolates were found in bovine mastitis milk samples. Of these, 74 colonies were from six conventional farms while 89 colonies were from seven organic farms. Further rapid coagulase test confirmed that only 17% (28 out of 163) exhibited coagulase activity, a characteristic of *S. aureus*. This included 15% (11) from four conventional dairy farms and 19% (17) from three organic dairy farms.

### 2.2. Antimicrobial Resistance of Coagulase-Positive Staphylococci (CoPS) and Presumptive S. aureus Isolates from Conventional and Organic Dairy Farms

The CoPS and potential *S. aureus* isolates from both conventional and organic dairy farms were tested for antimicrobial resistance against nine antimicrobial agents (Table 1).

Overall, the CoPS isolates exhibited less than 50% resistance against the following antibiotics used in this study: gentamicin (46.4%), tetracycline (46.4%), ampicillin (39.3%), erythromycin (21.4%), oxacillin (14.3%), and chloramphenicol (14.3%).

Table 1 showed that antibiotic resistance is more noticeable in conventional farm isolates than in organic farm isolates. Specifically, 81.8% of the conventional farm isolates exhibited resistance to ampicillin and tetracycline. Furthermore, resistance was observed in 45.5% of isolates for erythromycin, 36.4% for oxacillin, and 18.2% for chloramphenicol. Conversely, organic farm isolates exhibited lower resistance against tetracycline (23.5%), ampicillin (11.8%), chloramphenicol (11.8%), and erythromycin (5.9%). However, resistance to gentamicin was higher in organic farm isolates at 52.9% compared to conventional farm isolates (36.4%). 

**Figure 1:**
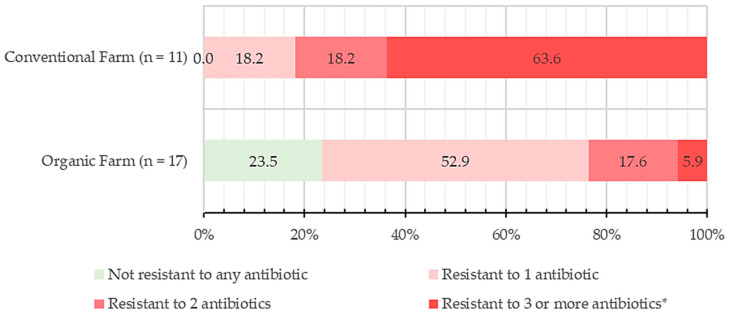
Percent isolate (%) classification of coagulase-positive *Staphylococcus* (CoPS) isolates from bovine mastitis milk in conventional and organic dairy farms according to the number of antibiotics resistant against (n = number of CoPS isolates tested). The antimicrobial agents tested were ampicillin, oxacillin, gentamicin, teicoplanin, vancomycin, erythromycin, chloramphenicol, ciprofloxacin, and tetracycline. The asterisk (*) in category denotes significant difference (*p* < 0.05) between conventional and organic farm samples.

All CoPS isolates were grouped and categorized based on their resistance to different numbers of antimicrobial agents (shown in Figure 1). All CoPS isolates from conventional farms exhibited resistance to at least one antibiotic. In contrast, 23.5% of the organic farm isolates did not show resistance to any tested antimicrobials in this study. The majority of CoPS isolates from conventional farms (63.6%) were resistant to three or more antibiotics, while 18.2% were resistant to two antibiotics, and an equal percentage showed resistance to only one antibiotic. Organic farm CoPS isolates primarily consisted of 52.9% showing resistance to one antibiotic and 17.6% resistant to two antimicrobials. A small fraction (5.9%) of organic farm isolates, specifically from OF2, demonstrated resistance to three or more antibiotics.

**Figure 2:**
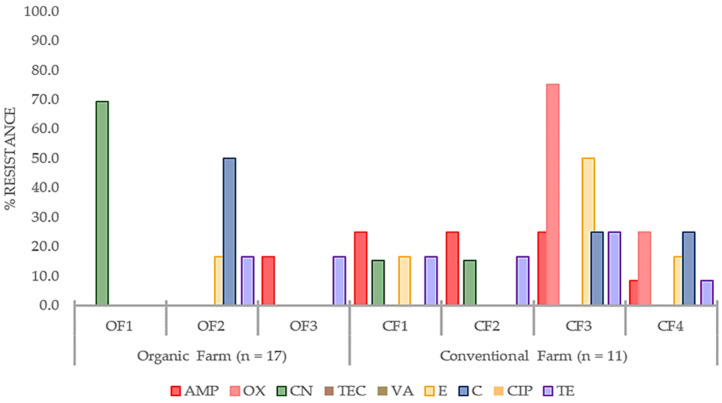
Relative antimicrobial resistance (%) of coagulase-positive *Staphylococci* (CoPS) isolated from bovine mastitis (BM) milk per dairy farm (n = number of CoPS isolates tested). Antibiotics tested were ampicillin (AMP), oxacillin (OX), gentamicin (CN), teicoplanin (TEC), vancomycin (VA), erythromycin (E), chloramphenicol (C), ciprofloxacin (CIP), and tetracycline (TE).

Figure 2 illustrates the comparative resistance of all coagulase-positive isolates to individual antimicrobials on each dairy farm. Each bar graph represent an antibiotic and every farm with multiple bars was likely to develop multi-drug resistance. As shown in the figure below, isolates from all conventional farms (CF1, CF2, CF3, and CF4) and one organic farm (OF2) exhibited resistance against three or more antimicrobial, particularly the β-lactams. Among conventional farm CoPS isolates, CF3 was observed with higher relative resistance, especially against oxacillin. Interestingly, organic farms have also exhibited resistance to different antibiotics, with OF1 having a higher relative resistance to gentamicin.

### 2.3. DNA Extraction and PCR Amplification of Isolated S. aureus Isolates

**Figure 3:**
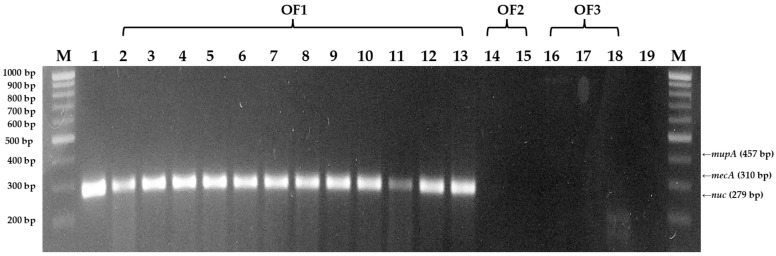
Agarose gel visualization of M-PCR detecting *mecA*, *mupA*, and *nuc* genes in bovine mastitis milk *S. aureus* isolates from organic farms (Lanes 2–18; OF1, OF2, OF3 are organic farms 1, 2, and 3, respectively) with negative template control (Lane 19; no dna template added), and *nuc* gene control *Staphylococcus aureus* (Lane 1).

**Figure 4:**
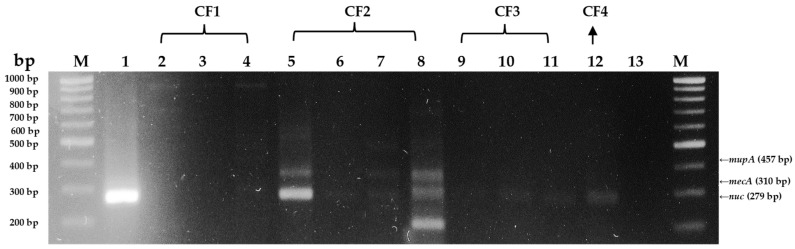
Agarose gel visualization of M-PCR detecting *mecA*, *mupA*, and *nuc* genes in bovine mastitis milk *S. aureus* isolates from conventional farms (Lanes 2–12; CF1, CF2, CF3, CF4 are conventional farms 1, 2, 3, and 4, respectively) with negative template control (Lane 13; no dna template added), and *nuc* gene control *Staphylococcus aureus* (Lane 1).

DNA of *S. aureus* isolates were extracted using a bacterial DNA extraction kit and subjected to PCR amplification. The corresponding agarose gel visualization confirmed which isolates from organic (Figure 3) and conventional (Figure 4) farms contained the *nuc*, *mecA*, and *mupA* genes.

Of the 3 organic farm samples, all 12 isolates from the organic farm 1, OF1, contained the *nuc* gene. Meanwhile, at least one isolate from conventional farms, CF2, CF3, and CF4, contained the *nuc* gene. Additionally, the *mecA* gene was found in three isolates from conventional farm 2 (CF2) but not in any of the organic farm isolates. On the other hand, the *mupA* gene was not detected in any of the isolates from both conventional and organic dairy farms.

## 3. Discussion

In this study, higher counts of suspected *S. aureus* were found in BM milk from organic farms (89 isolates) compared to conventional farms (74 isolates). This finding aligns with previous studies by Tikofsky et al. [10], Roesch et al. [15], and Sato et al. [16], who also reported higher *S. aureus* counts in organic farms in the US, Switzerland, and Wisconsin, respectively. However, in Denmark, dairy herds exhibited a contrasting pattern, with conventional farms displaying a slightly higher count of *S. aureus* isolates (77) compared to organic farms (75) [16]. The higher *S. aureus* count found in organic farms could be attributed to their very limited use of antibiotics, which are only used in emergency cases to save the cattle’s life [10,11]. The prohibited use of non-therapeutic antibiotics and growth promoters in organic farms, except in critical situations where animal safety is compromised [10,11,17] could result in abundant and diverse microbiota. This means organic farms could have higher microbial count and varied microbial species compared to conventional farms. Even so, CoPS isolates were found in more than half of the conventional farm samples (four out of six) compared to organic farm samples (three out of seven). This result was confirmed by M-PCR amplification and shown through the agarose gel electrophoresis, where some organic farm milk isolates did not contain the *nuc* gene marker of *S. aureus* but were positive for coagulase activity. The restricted use of antimicrobials in organic farms could explain the presence of more varied species of microorganisms thriving in organic farms [10,15,16,17].

Antimicrobial susceptibility testing revealed that conventional CoPS isolates showed significantly higher resistance to ampicillin, oxacillin, erythromycin, and tetracycline compared to organic CoPS isolates. Similar trends were observed in a study on MRSA from organic and conventional herds in Germany, where conventional farm isolates exhibited higher resistance to penicillin (100%), cefoxitin (100%), tetracycline (97.2%), erythromycin (44.4%), gentamicin (11.1%), and chloramphenicol (8.3%) [18]. Although a different bacterial species, Ray et al. [19] also found that conventional farm isolates of *Salmonella* from milk samples were more resistant to streptomycin and sulfamethoxazole.

Antibiotic use has long been linked to the subsequent rise of antimicrobial resistance, where resistance intensifies or accumulates with each antibiotic exposure due to selective pressure [20,21,22]. The most widely used antimicrobials for treating mastitis are β-lactam compounds such as ampicillin and oxacillin [2,23,24], which are also used in Korea [12,25]. The high resistance to antibiotics observed in this study could also be attributed to the fact that they are also commonly used to treat other diseases, aside from mastitis, in conventional dairy farms [12,25]. The consistent exposure to these antibiotics could potentially contribute to the increased antibiotic resistance observed in the CoPS isolates in this study. It is important to note that the chance of developing resistance is directly proportional to the amount of antibiotics used, as well as the frequency of antibiotic use [10,20].

Meanwhile, the high gentamicin resistance observed in CoPS from organic dairy farms could be attributed to its use as a veterinary drug for diarrhea treatment [26]. This result differs from the 2020 nationwide monitoring, which reported no gentamicin resistance in cattle-isolated *S. aureus* [12]. This finding suggests an increase in gentamicin usage in farms, particularly in organic cattle farms. The 2022 nationwide monitoring report confirmed an increase in gentamicin resistance in livestock farms [25], which supports our observation. This warrants further investigation and close monitoring on the allowed antibiotics that are used in emergency situations in organic farms in Korea.

Erythromycin and tetracycline are not prohibited in the cattle livestock industry in Korea, but similar compounds like spiramycin and tylosin (macrolides), and chlortetracycline and oxytetracycline (tetracyclines) are used instead [12,25]. The topmost sold antibiotic classes for livestock and fisheries in Korea, in descending order, are: β-lactams, tetracyclines, phenicols, macrolides, sulfonamides, and aminoglycosides [12,25].

Although resistance levels were less than 50% for most antibiotics, the implication of multidrug resistance was evident in CoPS isolates from both farm types. More multi-drug resistant CoPS isolates were found in conventional farms than in organic farms. As shown in Figure 2, the CoPS isolates with resistance to three or more antibiotic classes were classified as “multidrug-resistant” [27]. This could also be attributed to the regular antibiotic exposure of infected cattle in conventional farms as a BM treatment method [18,22]. As mentioned earlier, the amount and frequency of antibiotic use is directly related to the rate of developing antibiotic resistance [10,20]. However, exposure parameters like dosage, administration route, and dose frequency, were not specified, as antibiotic usage is difficult to monitor and quantify, especially in conventional farms [17]. Grobbel et al. [27] found that conventional farm isolates were resistant to three or more antibiotics when investigating antimicrobial resistance of *Escherichia coli* from organic and conventional poultry farms in Germany. Conversely, Meissner et al. [11] reported a similar percentage of multidrug-resistant isolates of extended-spectrum β-lactamase-producing *E. coli* from conventional and organic pig fattening farms.

Isolates from organic farm 1 (OF 1) were confirmed to contain the *nuc* gene, a specific marker for *S. aureus* [7,28]. In contrast, isolates from conventional farm 1 (CF1) and organic farms 2 and 3 (OF2 and OF3), lacking the *nuc* gene, may belong to other CoPS species. These isolates need further identification tests. Other *Staphylococcus* spp., including *S. intermedius*, *S. schleiferi* subsp. *coagulans*, *S. hyicus*, *S. lutrae*, *S. delhini*, and *S. pseudintermedius*, also demonstrate coagulase activity, antibiotic resistance, and biofilm formation [29,30]. These CoPS are opportunistic pathogens with potent virulence factors that can easily adapt according to their surroundings or hosts [29].

Three isolates from CF2 were confirmed to have the *mecA* gene, a specific marker for MRSA and a known determining factor of methicillin resistance [7,31]. This finding aligns with their observed corresponding antimicrobial resistance. The *mecA*-positive isolates from CF2 exhibited resistance to ampicillin and oxacillin (Figure 2). However, the *mupA* gene, a determinant of mupirocin resistance [7,30], was not detected in any isolates from the conventional and organic farms. Mupirocin, an antibiotic that inhibits bacterial synthesis, is highly effective against *S. aureus* [8,32]. It acts by binding to the enzyme isoleucyl-transfer RNA synthetase, preventing isoleucine incorporation during bacterial protein synthesis, making it effective against MRSA and some methicillin-resistant CoNS [7,8,32].

The investigation for mupirocin-resistant isolates aimed to detect any potential inter-host-adapted (humans to cattle and vice versa) species of CoPS and *S. aureus*. Different serovars of host-adapted *S. aureus* could arise through host-switching and gene acquisition, loss, and diversification [5]. The absence of *mupA* gene in all isolates suggests the higher effectivity of mupirocin and its related compounds as an antibiotic against CoPS and *S. aureus* isolated from conventional and organic dairy farms in South Korea. However, strict monitoring and regulation of mupirocin use and related substances are necessary.

## 4. Materials and Methods

Tryptic soy broth (TSB) and Baird–Parker agar (BPA) were purchased from BD Difco™ (Franklin Lakes, NJ, USA). Egg-yolk tellurite emulsion was bought from Oxoid (New Hampshire, UK). Muller–Hinton agar (MHA), nutrient agar (NA), and nutrient broth (NB) were distributed by MBCell (Seoul, Republic of Korea). Antibiotics were used; ampicillin (AMP) was obtained from GA BioChem (Atlanta, GA, USA) while oxacillin (OX), gentamicin (CN), tetracycline (TE), erythromycin (E), chloramphenicol (C), ciprofloxacin (CIP), vancomycin (VA), and teicoplanin, (TEC) were supplied by LiofilChem^®^ (Roseto degli Abruzzi, Italy). Pastorex Staph-plus latex agglutination test kit was from Bio-Rad (Hercules, CA, USA). PCR premix and oligo*nuc*leotide primers were secured from Bioneer (Daejeon, Republic of Korea). Bacterial DNA extraction kit was provided by Takara Bio (Shiga, Japan). Sodium chloride (NaCl) was ordered from Sigma-Aldrich (St. Louis, MO, USA). All other reagents, media, and materials used were HPLC or analytical grade.

### 4.1. Sample Collection and Isolation of S. aureus from Bovine Mastitis Milk Samples

Pooled raw milk samples from mastitis-infected (clinical and sub-clinical) cattle were aseptically collected from six conventional dairy farms and seven organic-certified dairy farms from the southern area of Gyeonggido and Chungcheongnamdo, South Korea, in late 2021 to the first quarter of 2022. The selection of participating dairy farm was based on their proximity to the university, with a preference to those that minimized sample travel time from the farm to the laboratory. Only farms that agreed to participate were included in this study.

Milk samples from cattle exhibiting signs of udder inflammation and abnormal milk (visible discoloration) were classified as clinical mastitis. On the other hand, milk samples with a high somatic cell count (>200,000 cells/mL) from cattle showing no clinical signs of mastitis were categorized as sub-clinical mastitis.

For each infected cattle, the teat surface was disinfected with 70% ethanol. After discarding a few streams of milk, 10 mL of milk was collected from each teat into sterile polyethylene screw-capped bottles. Milk samples from individual cattle were pooled per farm and kept in an icebox (at approximately 4 °C) and immediately transported to the laboratory for analysis.

*S. aureus* was isolated according to the Korean Food Codex [14]. For each sample, 25 g of milk was transferred in a sample filter bag and mixed with 225 mL of TSB containing 10% NaCl using stomacher (BagMixer 400CC, InterScience, Saint Nom, France). The homogenized mixture was then incubated at 36 ± 1 °C for 18–24 h. The enriched media was plated onto BPA supplemented with 5% egg-yolk tellurite emulsion and incubated at 36 ± 1 °C for another 18–24 h. Each black, glossy colony surrounded by transparent bands was separated and cultured to NA for further isolation. Gram-positive cocci with staphylococcal arrangement were verified using Gram staining. Further confirmation was carried out by rapid coagulase test using Pastorex™ Staph-plus latex agglutination test kit (Bio-Rad, Hercules, CA, USA). Confirmed presumptive *S. aureus* cultures were stocked in sterile glycerol solution and stored at −20 °C until further analysis. Retesting and subculture onto NA were carried out whenever the storage period exceeded 7 days.

### 4.2. Antimicrobial Susceptibility Testing of Isolated S. aureus Cultures Using a Disc Diffusion Method

Antimicrobial susceptibility testing was carried out following the guidelines of the Clinical and Laboratory Standards Institute (CLSI) [33] using a disc diffusion test against nine antibiotics (ampicillin, oxacillin, gentamicin, teicoplanin, vancomycin, erythromycin, chloramphenicol, ciprofloxacin, and tetracycline) as described in Table 2 with their corresponding concentration and inhibition zones.

A bacterial suspension was made following the density of McFarland 0.5 turbidity standard (1.5 × 10^8^ CFU/mL) and swabbed evenly onto the entire surface of MHA plates. Antibiotic discs were applied firmly onto the inoculated MHA plate with enough space for the inhibition zones. The agar plates were then incubated at 36 ± 1 °C for 18–24 h and inhibition zones measured using vernier caliper. Isolates were tested for each antibiotic on duplicate plates and classified (resistant, intermediate, susceptible) according to the average of their corresponding inhibition zones.

### 4.3. Bacterial DNA Extraction, PCR Amplification, and Gel Electrophoresis

Glycerol stocks of isolated *S. aureus* samples were plated on NA and incubated at 36 ± 1 °C for 18–24 h. Bacterial DNA was obtained following Takara MiniBEST bacteria genomic DNA extraction kit (Takara Bio, Shiga, Japan). First, lysis was carried out by making 1 mL of a bacterial suspension (0.5–2.0 × 10^9^ CFU/mL) and centrifugation at 12,000 rpm for 2 min. The supernatant was discarded and 50 µL buffer BS and 50 µL lysozyme were added to the bacteria pellet. The mixture was homogenized and incubated in a 37 °C waterbath for 1 h, with occasional inversion mixing every 20 min. The incubated solution was centrifuged at 12,000 rpm for 5 min and supernatant was discarded. Then 180 µL buffer GL, 20 µL proteinase K, and 10 µL RNase A were added, mixed thoroughly, and incubated at 56 °C waterbath for 10–30 min, with pipet mixing every 5 min until the solution became clear. Buffer GB (200 µL) and 100% ethanol (200 µL) were added and mixed well. The solution was then transferred to the spin column with a collection tube and centrifuged at 12,000 rpm for 2 min. The flow-through was discarded and buffer WA (500 µL) was added to the spin column and centrifuged (12,000 rpm, 1 min). The flow-through was removed and the spin column was washed with 700 µL buffer WB and centrifuged (12,000 rpm, 1 min). The flow-through was discarded and spin column was washed with buffer WB once more and centrifuged (12,000 rpm, 2 min). Afterwards, the spin column was transferred to a new 1.5 mL centrifuge tube with 50 µL elution buffer and left to stand at room temperature for 5 min before centrifugation (12,000 rpm, 2 min) to elute the DNA.

Extracted DNA samples were then subjected to PCR amplification using the following primers (Table 3):

Multiplex PCR (M-PCR) assay was performed according to [7] with slight modifications. An aliquot of 1 µL bacterial DNA template was added to 20-µL PCR premix containing the reaction buffer with 1.5 mM MgCl2, 250 µM each of deoxynucleoside triphosphate (dATP, dUTP, dGTP, and dCTP), 1.0 U of top DNA polymerase (Bioneer, Daejeon, Republic of Korea), and 1 µL of each primer for *mecA* (1.2 µM), *nuc* (0.4 µM), and *mupA* (0.5 µM). Amplification was carried out using a Q-Cycler II gradient thermal cycler (QuantaBiotech, Surrey, UK) as follows: initial denaturation step at 94 °C for 5 min; 10 cycles of 94 °C for 40 s, 58 °C for 40 s, and 72 °C for 1 min; 25 cycles of 94 °C for 1 min, 50 °C for 1 min, and 72 °C for 2 min; and final extension step at 72 °C for 10 min. Amplified samples were stored at 4 °C prior to gel electrophoresis. PCR amplicons were loaded on 2.0% agarose gel (Bioneer, Daejeon, Republic of Korea) and tris acetate EDTA (TAE) buffer containing 0.5 µg/mL mango blue dye and visualized under a UV light box using digital image system (GDS-200D, MDM, Suwon, Republic of Korea).

### 4.4. Statistical Analysis

The connection between type of farm (conventional or organic) and isolate activity (% resistance) for each antibiotic were analyzed using Chi-Square analysis for independent variance with Statistical Packages for the Social Sciences (IBM SPSS Statistics 23.0) software (SPSS Inc., Chicago, IL, USA). Significant differences among data were identified with *p* < 0.05 values. The same treatment was conducted for the relationship between farm type and resistant categories.

## 5. Conclusions

This study revealed variations in the prevalence of CoPS and *S. aureus* isolates from mastitis-infected cow’s milk across organic and conventional dairy farms in South Korea. Organic farms had a higher isolate count, but these isolates showed low antimicrobial resistance and lacked the *mecA* gene. On the other hand, isolates from conventional farms demonstrated higher resistance to antibiotics, particularly ampicillin and tetracycline, and were found to contain the *mecA* gene. The observations of this study showed the prospect of preventing or minimizing antimicrobial resistance and prevalence of MRSA bacterial strains using organic farming. Additionally, these findings also indicated the potential risk of developing multidrug resistance in bovine mastitis and livestock-associated bacterial isolates from dairy farms in South Korea, specifically in conventional dairy farms where monitoring and regulation of antibiotic use pose significant challenges. This highlights the need for a more comprehensive surveillance system and stricter rules and regulations for antibiotic use in conventional dairy farms to prevent potential future occurrences of multidrug resistance. Further analyses are required to identify other *Staphylococcus* species among isolates that do not carry the *mecA*, *mupA*, or *nuc* gene.

## Figures and Tables

**Table 1:** Antimicrobial resistance (number and % of resistant isolate) of coagulase-positive *Staphylococcus* from conventional and organic farms.

Antibiotic Name	Code	Conventional Farm (n = 11)		Organic Farm (n = 17)		Total (n = 28)	
		Number	%	Number	%	Number	%
Ampicillin	AMP	9	81.8 ^b^	2	11.8 ^a^	11	39.3
Oxacillin	OX	4	36.4 ^b^	0	0.0 ^a^	4	14.3
Gentamicin	CN	4	36.4 ^a^	9	52.9 ^a^	13	46.4
Teicoplanin	TEC	0	0.0 ^a^	0	0.0 ^a^	0	0.0
Vancomycin	VA	0	0.0 ^a^	0	0.0 ^a^	0	0.0
Erythromycin	E	5	45.5 ^b^	1	5.9 ^a^	6	21.4
Chloramphenicol	C	2	18.2 ^a^	2	11.8 ^a^	4	14.3
Ciprofloxacin	CIP	0	0.0 ^a^	0	0.0 ^a^	0	0.0
Tetracycline	TE	9	81.8 ^b^	4	23.5 ^a^	13	46.4

Different superscript letters between columns denote a significant difference (*p* < 0.05) between conventional and organic farm samples based on Chi-square test.

**Table 2:** Class, concentrations, and inhibition zones of antibiotics that were used in this study.

Compound Class	Antibiotic Name	Code	Disc Concentration (μg)	Zone Diameter (mm)		
				R ^†^	I ^†^	S ^†^
β-lactams	Ampicillin	AMP	10	15	16–21	22
	Oxacillin	OX	10	17	18–24	25
Aminoglycosides	Gentamicin	CN	10	18	19–27	28
Glycopeptides	Teicoplanin	TEC	30	14	15–21	22
	Vancomycin	VA	30	16	17–21	22
Macrolides	Erythromycin	E	15	21	22–30	31
Phenicols	Chloramphenicol	C	10	18	19–26	27
Quinolones	Ciprofloxacin	CIP	5	21	22–30	31
Tetracyclines	Tetracycline	TE	30	23	24–30	31

^†^ R, I, and S mean resistant, intermediate, and susceptible, respectively.

**Table 3:** Primers used in PCR amplification of *S. aureus* isolates from bovine mastitis milk in conventional and organic farms.

Target Gene	Primer	Oligonucleotide Sequence (5′–3′)	Amplicon Size (bp)
*mecA*	*mecA* 1	GTAGAAATGACTGAACGTCCGATAA	310
	*mecA* 2	CCAATTCCACATTGTTTCGGTCTAA	
*nuc*	*nuc* 1	GCGATTGATGGTGATACGGTT	279
	*nuc* 2	AGCCAAGCCTTGACGAACTAAAGC	
*mupA*	*mupA* 1	TATATTATGCGATGGAAGGTTGG	457
	*mupA* 2	AATAAAATCAGCTGGAAAGTGTTG	

## Data Availability

Data are contained within the article.
